# Assessing the knowledge, attitude, and practice of Egyptian pharmacists on probiotics: a cross-sectional study

**DOI:** 10.1186/s12906-026-05406-2

**Published:** 2026-05-25

**Authors:** Noha S. El Baghdady, Mohamed M. Elmazar, Marwa S. Hamza

**Affiliations:** 1 Clinical Pharmacy Practice Department, Faculty of Pharmacy, The British University in Egypt, El-Sherouk City, Cairo Egypt; 2 Pharmacology and Biochemistry Department, Faculty of Pharmacy, The British University in Egypt, El-Sherouk City, Cairo Egypt

**Keywords:** Probiotics, Knowledge, Attitude, Practice, Pharmacists, Egypt

## Abstract

**Background:**

Probiotics, live microorganisms offering health benefits, have gained attention for their potential in preventing various health conditions. Despite their availability, usage remains limited, possibly due to a lack of understanding among healthcare professionals. This study assesses the knowledge, attitude, and practice (KAP) of Egyptian pharmacists regarding probiotics.

**Methods:**

A descriptive, cross-sectional study was conducted using a self-administered questionnaire to evaluate the KAP of Egyptian pharmacists concerning probiotics. Data were collected using a convenient sampling method. The questionnaire, developed after a literature review and pilot-tested for reliability, included sections on demographics, knowledge, attitudes, practices, and barriers to recommending probiotics.

**Results:**

Only 11% of pharmacists demonstrated good knowledge of probiotics, while 82% exhibited a positive attitude. However, just 3% reported good practice in recommending probiotics. Younger pharmacists (18–24 years) and those with less experience (0–4 years) showed poorer knowledge and attitudes. In contrast, older pharmacists (45–54 years) and those with more experience (10–14 years) had better knowledge and attitudes. Female pharmacists were more likely to have a positive attitude than males. Barriers to recommending probiotics included a lack of established clinical applications and insufficient knowledge.

**Conclusion:**

The study highlights significant gaps in the knowledge and practice of Egyptian pharmacists regarding probiotics, despite positive attitudes. Age, experience, and professional exposure are crucial factors influencing KAP. Enhanced educational resources and training are needed to improve pharmacists’ understanding and integration of probiotics into healthcare practices.

**Supplementary Information:**

The online version contains supplementary material available at 10.1186/s12906-026-05406-2.

## Background

In recent years, probiotics have attracted substantial interest due to their potential health benefits [1]. Although the most frequently utilized microorganisms as probiotics belong to the Lactobacillus and Bifidobacterium genera, other bacterial genera such as Enterococcus, Streptococcus, and Escherichia are also employed. Additionally, the fungus Saccharomyces boulardii is recognized as a probiotic [2]. The health-promoting effects of probiotics include the maintenance of a balanced intestinal microbiota, modulation of the immune system, enhancement of metabolic health, and potential benefits in managing conditions such as obesity, type 2 diabetes, osteoporosis, and irritable bowel syndrome. Probiotics have also been shown to support gut homeostasis, reduce inflammation, and improve nutrient absorption [3]. Many previous clinical trials have demonstrated that probiotic strains are both safe and effective in delivering a range of health benefits. These benefits encompass the prevention of cardiovascular and urogenital infections, cancer, lactose intolerance, cystic fibrosis, and various oral diseases. Furthermore, probiotics may contribute to the prevention of tooth decay, the treatment of periodontal disease, the reduction of oral halitosis [4], and preventing antibiotic-associated diarrhea is one of the most common use of probiotics [5]. Probiotics are available in the market in pharmaceutical formulations and functional foods [6].

Egypt’s probiotics market is expected to increase at a compound annual growth rate of 7.82%, reaching US$225.741 million by 2029, up from US$132.042 million in 2022. Strategic alliances and mergers are becoming more common in Egypt’s probiotics business, which is helping to drive growth [7]. Despite the availability of a diverse selection of probiotic products on the market and the abundance of evidence published over the last ten years indicating the benefits of probiotics for health, their usage is not as prevalent as that of other supplements. This could be due to lack of understanding and a shortage of health-care experts who can provide public health information [8]. Probiotics have limitations due to potential side effects, which can include gastrointestinal discomfort, infections in immunocompromised individuals, and adverse reactions in some patients. More awareness and research are required in this probiotics field to understand the proper guidance and limits [9]. Another factors contributing to their limited use include variability in strain-specific efficacy and safety, lack of robust evidence for certain products, and the need for careful evaluation of manufacturing practices [10]. Furthermore, healthcare personnel’ understanding of probiotics has a direct impact on the therapy choice and outcome [6].

Pharmacists’ knowledge, attitudes, and practices (KAP) regarding probiotics vary significantly across different regions and contexts. While pharmacists generally exhibit a positive attitude towards probiotics, their knowledge and practices often reveal gaps, particularly concerning the broader health benefits of probiotics beyond digestive health. This discrepancy highlights the need for targeted educational interventions to enhance pharmacists’ understanding and application of probiotics in healthcare settings. In Pakistan, pharmacists showed a significant association with good knowledge about probiotics (89.1%). However, overall knowledge among healthcare professionals was poor, with only 15.1% demonstrating good knowledge [11]. In the UAE, while 91.2% of pharmacists recognized the role of probiotics in immune support, only 30% were aware of their cardiovascular benefits, indicating a gap in knowledge about the broader applications of probiotics [12]. In practice, pharmacists in the UAE often recommend storing probiotics at room temperature, and misconceptions about probiotics being primarily for gastrointestinal issues are common [12]. Pharmacy students in Türkiye also demonstrated a strong belief in the health benefits of probiotics, with 91.9% acknowledging their positive impact [13]. In Turkey, pharmacists frequently recommend probiotics for gastrointestinal disorders, with 99.7% doing so, and are more likely than physicians to suggest them for genitourinary issues [14].

Despite the positive attitudes, the knowledge gaps and misconceptions about probiotics’ applications beyond digestive health suggest a need for enhanced educational efforts. Addressing these gaps through professional development and training can empower pharmacists to better educate patients and promote the comprehensive benefits of probiotics.

Many studies were conducted to evaluate knowledge, attitudes, and practice patterns of healthcare providers (HCPs) toward probiotics in different countries [1, 11, 14–16]. However, up to our knowledge no studies have been conducted in Egypt. This study aims to study the Egyptian pharmacists’ knowledge, attitudes, and practices concerning probiotics, exploring potential barriers that may hinder their incorporation of probiotics into patient care.

## Methods

### Study design

The present study is a descriptive, cross-sectional study. It was carried out using a self-administered questionnaire to evaluate the KAP of Egyptian pharmacists concerning probiotics use. Data collection was performed using a convenient sampling method from June 2024 to August 2024. Any incomplete responses were omitted from the final study results.

### Sample size & response rate

A sample size of 385 was determined using the Raosoft sample size calculator, based on a 95% confidence interval (CI), a 50% response distribution, a Z-score of 1.96, and a 5% margin of error. This approach ensures that the study findings are statistically representative of the target population, minimizing the risk of sampling bias. To account for potential response errors, missing data, and non-response bias, we increased the target sample size by approximately 10%. This study employed a convenience sampling method, allowing for the inclusion of pharmacists from diverse practice settings, including, community, hospital, academic, and clinical pharmacy roles as well as medical representatives. A total of 435 surveys were collected, of which 76 responses were excluded due to incompleteness, resulting in a final sample of 347 valid responses. The overall response rate was 80% (347/435).

### Questionnaire development

A pre-validated questionnaire [11] was developed and modified according to the need after conducting an extensive literature review [11, 17, 18]. The questionnaire’s clarity and utility were initially assessed through a review process involving five pharmacist specialists from different fields: Community Pharmacy, Hospital Pharmacy, Sales and Marketing, Academia, and the Pharmaceutical Industry. Their suggested modifications were subsequently incorporated to ensure the questionnaire’s relevance across various pharmacy practice settings. Subsequently, a pilot study with 10 participants was conducted to examine the questionnaire’s reliability and validity, yielding a Cronbach’s alpha of 0.75, which indicated satisfactory reliability. The finalized questionnaire was structured into five distinct sections, with the first section focusing on demographic data. The second section was dedicated to evaluating knowledge about probiotics, comprising 10 questions designed to measure respondents’ comprehension of probiotics. These questions were designed to get the most accurate definition of probiotics, identify recognized strains, assess perceived health benefits, and determine awareness of dietary sources and commercial products. A scoring system was applied where each correct answer was assigned a score of 1, while incorrect or unanswered responses received a score of 0. For questions utilizing a three-point Likert scale, both “No” and “I do not know” responses were scored as zero. This scoring method was chosen because both responses indicate a lack of correct knowledge rather than partial understanding. Assigning the same score ensures consistency in evaluating pharmacists’ knowledge, as uncertainty (“I do not know”) functionally equates to an incorrect answer (“No”) when assessing factual information. This approach has been commonly applied in previous KAP studies to distinguish between participants who possess knowledge and those who do not [19–21]. The maximum attainable score for this section was 42 points, with a score below 21 classified as poor knowledge, and a score of 21 or above indicative of good knowledge regarding probiotics. This cutoff was determined using the 50% threshold approach, a widely used method in KAP studies to differentiate between sufficient and insufficient knowledge levels [22]. By setting the midpoint as the benchmark, we ensure a clear and meaningful classification of respondents. Moreover, the cutoff point was validated during the questionnaire’s pilot testing phase to confirm its effectiveness in distinguishing between participants with adequate knowledge and those with gaps in understanding.

The third section of the survey comprises three items aimed at assessing pharmacists’ attitudes toward probiotics. The scoring system for this section utilizes a Likert scale, with responses to questions 1 and 3 scored as follows: “Not at all” is assigned 0 points, “Somewhat” is assigned 1 point, and “Very much” is assigned 2 points. Conversely, question 2 is an inverted item, such that the scoring is reversed: “Not at all” is assigned 3 points, and “Very much” is assigned 0 points. The maximum attainable attitude score is 6. Participants achieving a score greater than or equal to 3 are deemed to possess a positive attitude toward probiotics. The full questionnaire is available as Supplementary (1).

The fourth section of the survey is evaluating pharmacists’ practices concerning the recommendation of probiotics. This section aims to understand how pharmacists incorporate probiotics into their professional practice, including their recommendations for various health conditions and their views on the potential benefits of probiotics. Each correct response in this section is awarded a score of 1, while incorrect answers receive a score of 0. The total possible score for this section is 24. Participants who achieve a score of 12 or more are considered to demonstrate good practice in prescribing probiotics. The last section is designed to identify and explore the barriers that pharmacists face when it comes to prescribing probiotics.

### Ethical considerations

The Research Ethics committee at the Faculty of Pharmacy in the British University in Egypt has approved the conduct of the present study (CL-2403). In accordance with the institution’s established standards, all ethical principles were followed. To ensure anonymity, no personal information (such as names or IDs) was collected. Before agreeing to participate in the survey, all participants were properly informed about the study’s objectives and scope, and they guaranteed full anonymity and confidentiality. Each participant provided a digital informed consent. To avoid repeated submissions from the same person, we recorded each participant’s IP address and only one response per IP address was permitted (a feature enforced by Survey Monkey). Lastly, all participants were reminded that their involvement in the survey was completely voluntary.

### Statistical analysis

In the current investigation, descriptive and inferential statistical analyses were conducted utilizing SPSS Version 23.0 (IBM). Descriptive statistics involved the employment of frequencies and percentages to depict categorical variables. The Chi-square analysis with Fisher’s exact test were employed to examine the associations between Knowledge, Attitudes, and Practices (KAP) across different subgroups within the study. Binary logistic regression models were utilized to identify potential determinants of participants’ good knowledge, positive attitudes, and good practices concerning probiotics. The results of the regression analyses were presented using Adjusted Odds Crude Odds Ratios (COR) and Adjusted Odds Ratios (AOR), along with their corresponding 95% Confidence Intervals (CI), were calculated to assess the strength of associations between variables. COR represents the unadjusted relationship between an independent variable and the outcome, while AOR accounts for potential confounders by adjusting for covariates, including gender, age, profession, and years of experience. The logistic regression model included gender, age, profession, and years of experience, as these variables were selected based on previous literature [11, 14, 23], theoretical relevance, and their potential influence on pharmacists’ knowledge, attitudes, and practices regarding probiotics. Adjusting for these covariates ensures a more accurate and unbiased analysis of the factors influencing pharmacists’ KAP regarding probiotics. This adjustment provides a more precise estimate of the independent effect of each variable. A significance level of *p* < 0.05 was used for all statistical tests, indicating statistical significance when the p-value was below this threshold.

## Results

### Demographic characteristics of the study population

The study included 348 pharmacists, with a gender distribution of 53.6% male and 46.4% female. Most participants were aged between 18 and 34 years (76.3%), and most held a bachelor’s degree (69.3%). The largest professional roles were community pharmacist (39%) and clinical pharmacist (16.3%). The distribution of years of experience varied, with the majority having 0–4 years (59%) (Table 1).

**Table 1:** Demographic Data

	Frequency	Percent
Gender		
Male	187	53.6
Female	162	46.4
Age Group		
18–24	107	30.7
25–34	159	45.6
35–44	60	17.2
45–54	15	4.3
55–64	7	2.0
65+	1	0.3
Educational Background		
Bachelor’s degree	242	69.3
Post graduate diploma	39	11.2
Master’s degree	33	9.5
Ph.D.	35	10.0
Professional Role in the pharmacy field		
Hospital Pharmacist	47	13.5
Academic staff	42	12.0
Sales and Marketing	27	7.7
Research	11	3.2
Industry	12	3.4
Community Pharmacist	136	39.0
Senior pharmacy student (Internship year)	17	4.9
Clinical pharmacist	57	16.3
Years of experience		
0–4	206	59.0
5–9	67	19.2
10–14	36	10
15+	40	11
KAP results		
Good Knowledge	39	11
High Attitude	285	82
Good Practice	10	3

## Knowledge, attitude, and practice regarding probiotics

**Fig. 1:**
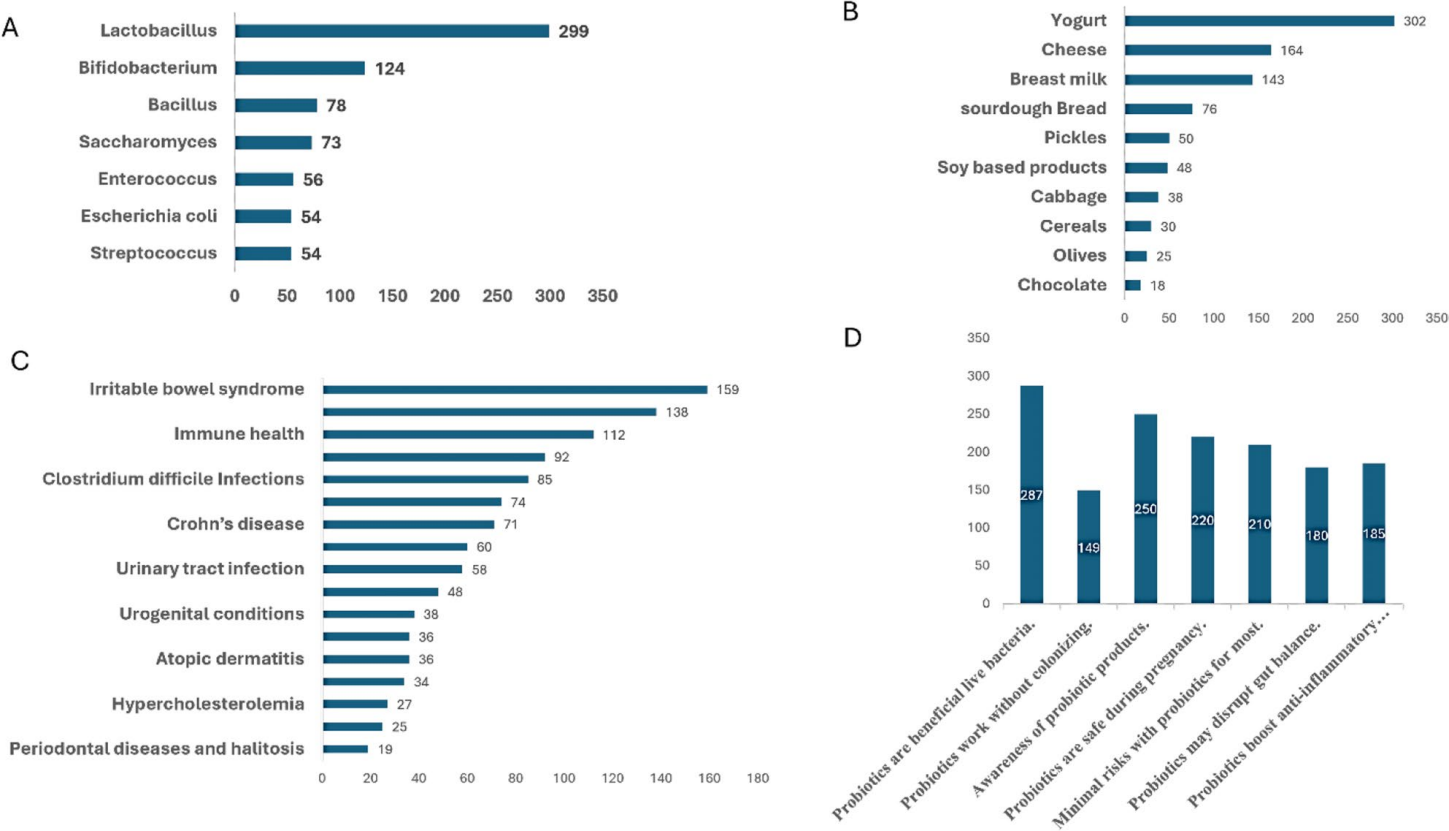
Probiotic Knowledge Insights among participants. **A** The strains of probiotics. **B** The dietary sources of probiotics. **C** Health Conditions Treatable with Probiotics. **D** Knowledge about correct definition of Probiotics

Table 1 also indicates that merely 11% of the pharmacists surveyed possess a comprehensive understanding of probiotics. For instance, Fig. 1 illustrates various probiotic strains and their corresponding health benefits, with the most pharmacists, 159, identifying IBS. The least number of pharmacists, 19, pointed out periodontal diseases and halitosis. When it comes to dietary sources of probiotics, the most cited by pharmacists was yogurt, with 302 pharmacists mentioning it.

**Fig. 2:**
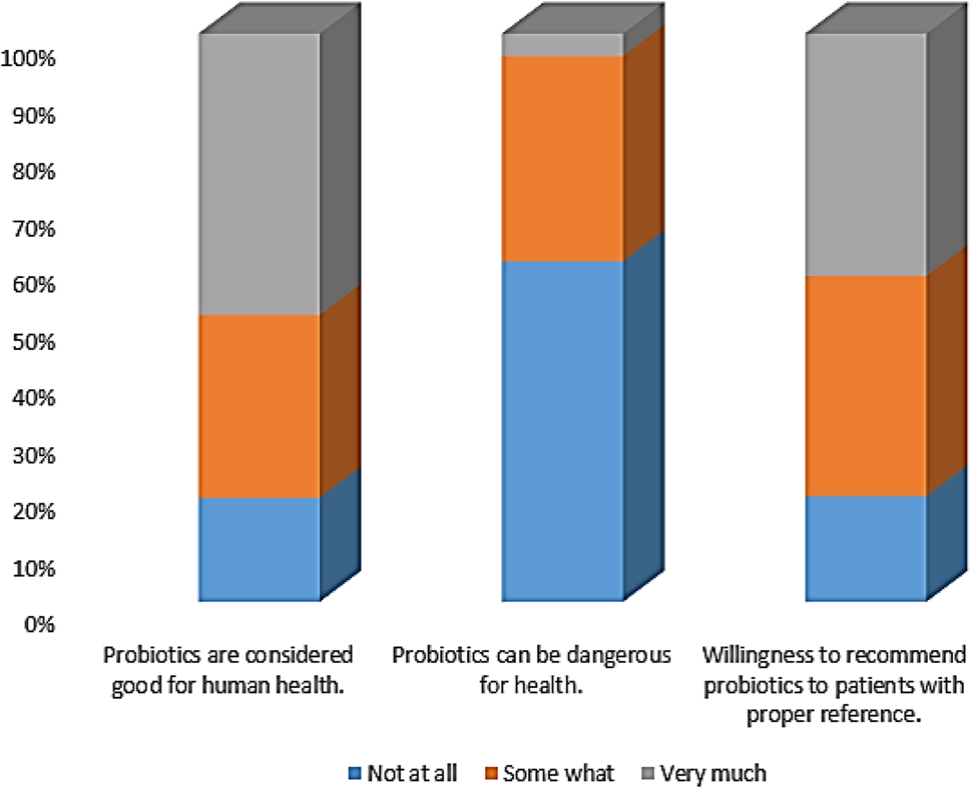
Participants attitude towards probiotics

**Fig. 3:**
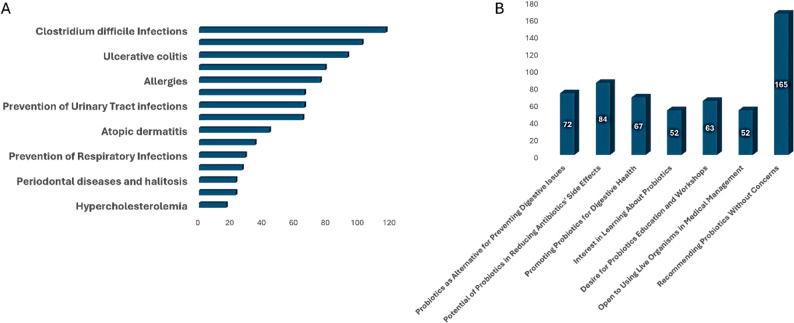
Participants’ Practices and Perceptions towards Probiotics. **A** Practice toward different health conditions. **B** Practices toward different aspects related to probiotics

Despite this, the majority, 82%, of pharmacists display a favorable disposition toward probiotics. Figure 2 depicts the attitudes toward probiotics and the willingness to endorse them, with the highest level of agreement among pharmacists being that probiotics are beneficial for health, at 100%. The lowest level of agreement was that probiotics could be harmful to health, with 0% agreement. In contrast to these positive attitudes, only 3% of pharmacists demonstrated good practice in recommending and using probiotics. Specific health conditions and pharmacists’ practices/perceptions regarding probiotics are detailed in Fig. 3, which reveals that the most common practice among pharmacists was in preventing urinary tract infections, with 165 pharmacists reporting this, and the least common practice was in preventing respiratory infections, with 20 pharmacists reporting this.

Table 2 provides a detailed comparison of pharmacists’ demographics and their corresponding knowledge, attitude, and practice regarding probiotics. The variables analyzed include gender, age group, educational background, professional role in the pharmacy field, and years of experience. Male pharmacists exhibited a high percentage of poor knowledge (90.4%) compared to females (87%), with a significant difference in attitude (*p* < 0.001) but not in practice (*p* ≥ 0.05). Males had a higher frequency of negative attitudes (25%) than females (10.5%). Both genders showed a high percentage of poor practice, with males at 96.8% and females at 97.5%. Younger pharmacists aged 18–24 had the highest percentage of poor knowledge (94.4%). Pharmacists aged 18–24 also showed the most negative attitude (39.3% negative), with those aged 45–54 and above having 100% positive attitudes with a significant differences in attitude were observed (*p* < 0.001). Poor practice was highly prevalent across all age groups, reaching 100% in the 35–44 and ≥ 65 age groups. Significant differences in practice were observed (*p* < 0.05).

**Table 2:** Difference in knowledge, attitude and practice about probiotics by demographics

Variable		Knowledge			Attitude			Practice		
		Poor	Good	*P* value	Negative	Positive	*P* value	Poor	Good	*P* value
Gender	Male	169 (90.4%)	18 (9.6%)	0.32	47 (25%)	140 (75%)	**< 0.001**	181 (96.8%)	6 (3.2%)	0.75
	Female	141 (87%)	21 (13%)		17 (10.5%)	145 (89.5%)		158 (97.5%)	4 (2.5%)	
Age	18–24	101 (94.4%)	6 (5.6%)	**0.03**	42 (39.3%)	65 (60.7%)	**< 0.001**	105 (98%)	2 (2%)	**0.04**
	25–34	141 (88.7%)	18 (11.3%)		14 (8.8%)	145 (91.2%)		154 (97%)	5 (3%)	
	35–44	51 (85%)	9 (15%)		7 (11.7%)	53 (88.3%)		60 (100%)	0 (0%)	
	45–54	10 (66.7%)	5 (33.3%)		0 (0%)	15 (100%)		13 (86.7%)	2 (13.3%)	
	55–64	6 (85.7%)	1 (14.3%)		1 (14.3%)	6 (85.7%)		6 (85.7%)	1 (14.3%)	
	65+	1 (100%)	0 (0%)		0 (0%)	1 (100%)		1 (100%)	0 (0%)	
Educational Background	Bachelor’s degree	220 (91%)	22 (9%)	0.13	52 (21.5%)	190 (78.5%)	**0.002**	235 (97%)	7 (3%)	0.4
	Post graduate diploma	35 (89.7%)	4 (10.3%)		9 (23%)	30 (77%)		39 (100%)	0 (0%)	
	Master’s degree	27 (81.8%)	6 (18.2%)		3 (9%)	30 (91%)		32 (97%)	1 (3%)	
	Ph.D.	28 (80%)	7 (20%)		0 (0%)	35 (100%)		33 (94.3%)	2 (5.7%)	
Current professional	Hospital Pharmacist	41 (87.2%)	6 (12.8%)	0.36	24 (51%	23 (49%)	**< 0.001**	44 (93.6%)	3 (6.4%)	0.4
	Academic staff	33 (78.6%)	9 (21.4%)		4 (9.5%)	38 (90.5%)		40 (95.2%)	2 (4.8%)	
	Sales and Marketing	25 (92.6%)	2 (7.4%)		2 (7.4%)	25 (92.6%)		26 (96.3%)	1 (3.7%)	
	Research	11 (100%)	0 (0%)		0 (0%)	11 (100%)		11 (100%)	0 (0%)	
	Industry	12 (100%)	0 (0%)		4 (33.3%)	8 (66.7%)		12 (100%)	0 (0%)	
	Community Pharmacist	123 (90.4%)	12 (9.6%)		27 (20%)	109 (80%)		134 (98.5%)	2 (1.5%)	
	Senior pharmacy student (Internship year)	16 (94.1%)	1 (5.9%)		0 (0%)	17 (100%)		16 (94%)	1 (6%)	
	Clinical pharmacist	49 (86%)	8 (14%)		3 (5.3%)	54 (94.7%)		56 (98.2%)	1 (1.8%)	
Years of experience	0–4	193 (93.7%)	13 (6.3%)	**0.004**	47 (22.8%)	159 (77.2%)	**0.007**	202 (98%)	4 (2%)	**0.04**
	5–9	57 (85%)	10 (15%)		12 (18%)	55 (82%)		65 (97%)	2 (3%)	
	10–14	28 (77.8%)	8 (22.2%)		4 (11%)	32 (89%)		36 (100%)	0 (0%)	
	15+	32 (80%)	8 (20%)		1 (2.5%)	39 (97.5%)		36 (90%)	4 (10%)	

*P* < 0.05 was considered to indicate significance. Bold fonts show significant differences

Pharmacists with a bachelor’s degree had the highest percentage of poor knowledge (91%) and negative attitude (21.5%), while those with a Ph.D. had the lowest percentage (80% and 0%, respectively). Significant differences in attitude (*p* < 0.05) were observed across educational backgrounds. Poor practice was almost universal across all educational levels. Sales and marketing professionals and clinical pharmacists had high positive attitudes (92.6% and 94.7% positive, respectively). Significant differences in attitude were observed (*p* < 0.001). Poor practice was highly prevalent across all professional roles, reaching 100% among researchers and industry pharmacists. Regarding years of experience, pharmacists with 0–4 years of experience had a high percentage of poor knowledge (93.7%) and negative attitude (22.8%) with significant differences observed across years of experience for both knowledge (*p* < 0.05) and attitude (*p* < 0.05)Those with 10–14 and 15 + years of experience showed a higher percentage of good knowledge (77.8% and 80%, respectively) and positive attitude (89% and 97.5%, respectively). Poor practice was almost universal across all experience levels, with the highest percentage among those with 10–14 years (100%).

### Binary logistic regression analysis for good knowledge of probiotics

Table 3 presents the binary logistic regression analysis for variables associated with good knowledge of probiotics among pharmacists. Pharmacists aged 45–54 were significantly associated with good knowledge. The COR for this age group was 8.417 (95% CI 2.2–32.5, *p* < 0.01), indicating a strong likelihood of good knowledge. After adjustment, the AOR was 2.71 (95% CI 0.32–55.7, *p* ≥ 0.05), suggesting that while the association was not as strong after considering other factors, there was still a trend indicating that age is a relevant factor in predicting good knowledge of probiotics. Having 10–14 years of experience was significantly associated with good knowledge. The COR for pharmacists with 10–14 years of experience was 4.2 (95% CI 1.6–11, *p* < 0.01), and the AOR remained significant at 4.3 (95% CI 1.1–15.6, *p* < 0.05), indicating an association between years of experience and good knowledge regarding probiotics.

**Table 3:** Binary logistic regression for variables related to good knowledge of pharmacist

Variable		Variables associated with good knowledge					
		COR	95% CI	*P* Value	AOR	95% CI	*P* Value
Gender	Male*						
	Female	1.4	0.7–2.7	0.32	1.28	0.6–2.7	0.5
Age	18–24*						
	25–34	2.149	0.8–5.6	0.11	1.74	0.6–5.1	0.31
	35–44	2.971	1.0-8.8	0.05	1.18	0.26–5.44	0.82
	45–54	8.417	2.2–32.5	**0.002**	2.71	0.32–55.7	0.35
	55–64	2.806	0.29–27.2	0.37	0.97	0.05–16.4	0.98
	65+	-	-	1	1.1	-	1.0
Educational Background	Bachelor’s degree*						
	Post graduate diploma	1.14	0.37–3.5	0.8	0.78	0.2–2.8	0.7
	Master’s degree	2.2	0.82–5.96	0.11	1.5	0.4–5.03	0.5
	Ph.D.	2.5	0.97–6.38	0.5	0.9	0.18–4.6	0.9
Current professional	Hospital Pharmacist*						
	Academic staff	1.8	0.6–5.7	2.8	0.83	0.17-4.0	0.8
	Sales and Marketing	0.5	0.1–2.9	4.8	0.39	0.06–2.2	0.3
	Research	0	-	0.9	0	-	0.9
	Industry	0	-	0.9	0	-	0.9
	Community Pharmacist	0.7	0.2-2.0	0.5	0.57	0.19–1.67	0.3
	Senior pharmacy student (Internship year)	0.4	0.04–3.8	0.4	0.55	0.05–5.7	0.6
	Clinical pharmacist	1.1	0.3–3.4	0.8	0.58	0.16–2.08	0.4
Years of experience	0–4*						
	5–9	2.6	1.08–6.2	**0.03**	2.38	0.8–6.4	0.08
	10–14	4.2	1.6–11	**0.003**	4.3	1.1–15.6	**0.03**
	15+	3.7	1.4–9.6	**0.007**	2.9	0.5–17.0	0.2

*P* < 0.05 was considered to indicate significance. Bold fonts show significant differences

*Indicates a reference group in the logistic regression

### Binary logistic regression analysis for positive attitude towards probiotics

Table 4 presents the binary logistic regression analysis for variables associated with a positive attitude toward probiotics among pharmacists. Being female was significantly associated with a positive attitude toward probiotics. The COR for females was 2.8 (95% CI 1.5–5.2, *p* < 0.001), indicating that female pharmacists were 2.8 times more likely to have a positive attitude compared to males. However, after adjustment, the AOR was 1.8 (95% CI 0.89–3.8, *p* < 0.05), suggesting that the difference in attitude by gender was not significant when other factors were considered, but the trend remained. Younger pharmacists aged 25–34 were significantly more likely to have a positive attitude toward probiotics. The COR for this age group was 6.7 (95% CI 3.4–13.1, *p* < 0.001), indicating a strong association. Even after adjustment, the AOR remained significant at 7.5 (95% CI 3.5–16.1, *p* < 0.001), confirming that age is a critical factor in predicting a positive attitude toward probiotics among pharmacists.

**Table 4:** Binary logistic regression for variables related to Positive attitude of pharmacist

Variable		Variables associated with Positive attitude					
		COR	95% CI	*P* Value	AOR	95% CI	*P* Value
Gender	Male*						
	Female	2.8	1.5–5.2	**0.001**	1.8	0.89–3.8	0.09
Age	18–24*						
	25–34	6.7	3.4–13.1	**< 0.001**	7.5	3.5–16.1	**< 0.001**
	35–44	4.9	2.0-11.7	**< 0.001**	3.5	0.8–14.8	0.07
	45–54	-	-	0.99	1.3	-	1
	55–64	3.8	0.45–33.3	0.21	0	-	0.9
	65+	-	-	1.00	0	-	0.9
Educational Background	Bachelor’s degree*						
	Post graduate diploma	0.9	0.4-2.0	0.8	0.5	0.18–1.6	0.26
	Master’s degree	2.7	0.8–9.3	0.1	0.7	0.14–4.1	0.76
	Ph.D.	-	-	0.9	-	-	0.9
Current professional	Hospital Pharmacist*						
	Academic staff	9.9	3.0-32.2	**< 0.001**	6.6	1.4–31.7	**0.01**
	Sales and Marketing	13	2.8–61.4	**0.001**	10.4	1.9–56.3	**0.006**
	Research	-	-	0.9	-	-	0.9
	Industry	2.08	0.5–7.8	0.27	1.5	0.27–8.2	0.6
	Community Pharmacist	4.2	2.0-8.5	**< 0.001**	3.8	1.6–8.6	**0.002**
	Senior pharmacy student (Internship year)	-	-	0.9	-	-	0.9
	Clinical pharmacist	18.7	5.1–68.6	**< 0.001**	13.8	3.2–59.8	**< 0.001**
Years of experience	0–4*						
	5–9	1.3	0.67–2.74	0.39	0.7	2.7–1.8	0.49
	10–14	2.3	0.79–7.02	0.12	1.6	0.33–7.89	0.54
	15+	11.5	1.54–86.1	**0.01**	-	-	0.99

*P* < 0.05 was considered to indicate significance. Bold fonts show significant differences

*Indicates a reference group in the logistic regression

Regarding the professional role, several professional roles were significantly associated with a positive attitude. Pharmacists working in sales and marketing had a COR of 13 (95% CI 2.8–61.4, *p* < 0.001), indicating a strong likelihood of a positive attitude. After adjustment, the AOR was 10.4 (95% CI 1.9–56.3, *p* < 0.05), which still suggests a significant association. Similarly, clinical pharmacists had a COR of 18.7 (95% CI 5.1–68.6, *p* < 0.001) and an AOR of 13.8 (95% CI 3.2–59.8, *p* < 0.001). In addition, community pharmacists were also significantly associated with a positive attitude, with a COR of 4.2 (95% CI 2.0–8.5, *p* < 0.001) and an AOR of 3.8 (95% CI 1.6–8.6, *p* = 0.002), indicating a robust and significant association with a positive attitude toward probiotics. No significant associations were found between educational background and positive attitude toward probiotics in the adjusted analysis. The analysis did not reveal significant associations between years of experience and positive attitude toward probiotics, except for pharmacists with 15 + years of experience, who had a COR of 11.5 (95% CI 1.54–86.1, *p* < 0.05), but this was not significant after adjustment as the value of AOR was considered as outlier, likely due to the small sample size in this category.

### Binary logistic regression analysis for poor practice towards probiotics

Table 5 presents the binary logistic regression analysis for variables associated with poor practice regarding probiotics among pharmacists. Pharmacists aged 45–54 were significantly associated with poor practice. The COR for this age group was 8 (95% CI 1.0-62.3, *p* < 0.05), indicating a strong likelihood of poor practice. After adjustment, the AOR was 0 (95% CI 0–0, *p* ≥ 0.05), suggesting that the difference in practice by age was not significant when other factors were considered. Pharmacists with 15 + years of experience had a significant association with poor practice. The COR for this group was 5.6 (95% CI 1.3-23.46, *p* < 0.05), and the AOR was an outlier value, which was likely due to the small sample size in this category.

**Table 5:** Binary logistic regression for variables related to Poor Practice of pharmacist

Variable		Variables associated with Good Practice					
		COR	95% CI	*P *Value	AOR	95% CI	*P *Value
Gender	Male*						
	Female	0.7	0.2–2.7	0.68	0.49	0.08–2.7	0.42
Age	18–24*						
	25–34	1.7	0.-8.9	0.50	6.3	0.5–69.0	**0.120**
	35–44	0	-	0.99	0	-	0.99
	45–54	8	1.0-62.3	**0.04**	0	-	0.99
	55–64	8.7	0.69–110	0.09	0	-	0.99
	65+	0	-	1	0	-	0.99
Educational Background	Bachelor’s degree*						
	Post graduate diploma	0	-	0.99	0	-	0.99
	Master’s degree	1	0.12–8.8	0.96	2.3	0.08–63.2	0.6
	Ph.D.	2	0.4–10.2	0.38	5.9	0.04–728.2	0.46
Current professional	Hospital Pharmacist*						
	Academic staff	0.73	0.1–4.6	0.74	0.02	0-4.6	**0.16**
	Sales and Marketing	0.56	0.05–5.7	0.62	0.16	0.01–2.3	**0.18**
	Research	0	-	0.99	0	-	0.99
	Industry	0	-	0.99	0	-	0.99
	Community Pharmacist	0.2	0.03–1.3	0.10	0.1	0.01–0.7	**0.02**
	Senior pharmacy student (Internship year)	0.9	0.08–9.4	0.9	0	-	0.99
	Clinical pharmacist	0.2	0.02–2.6	0.25	0.45	0.001-1.8	0.10
Years of experience	0–4*						
	5–9	1.5	0.2–8.6	6	2.1	0.3–14.7	0.45
	10–14	0	-	0.99	0	-	0.99
	15+	5.6	1.3-23.46	**0.01**	-	-	0.99

*P* < 0.05 was considered to indicate significance. Bold fonts show significant differences

*Indicates a reference group in the logistic regression

### Barriers for not recommending probiotics according to pharmacists

**Fig. 4:**
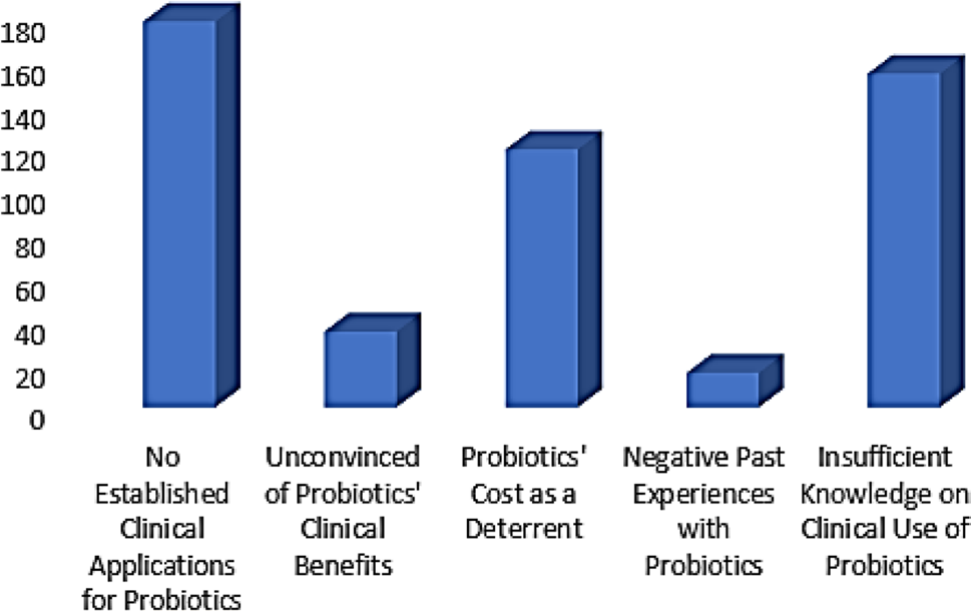
Barriers for not recommending probiotics

Figure 4 shows reasons for not recommending probiotics. Highest number of pharmacists’ concerns: No Established Clinical Applications for Probiotics with 200 pharmacists mentioning it. Lowest number of pharmacists’ concerns: Insufficient Knowledge on Clinical Use of Probiotics with 40 pharmacists reporting it.

## Discussion

The use of dietary and herbal supplements (DHSs) is prevalent worldwide [24, 25]. Probiotics serve as a prime example of dietary and herbal supplements. While probiotics were initially associated with gut health, recent research has broadened to investigate their role in preventing a range of other ailments. Evidence suggests that probiotics can bolster the immune system and are beneficial in managing conditions such as allergies, diarrhea, inflammatory bowel disease (IBD), irritable bowel syndrome (IBS), infections, infant colic, and certain cancers [26–28] but general public lacks awareness about the benefits of DHSs. Given that pharmacists often provide advice on DHSs to patients for various health conditions, it is essential for them to possess a solid understanding of these products.

The present study was aimed at assessing the KAP of Pharmacists regarding the use of probiotics and the barriers that are encountered in their prescribing. The results showed that only a small percentage of participants (11%) had good knowledge regarding probiotic use. These findings are not consistent with reports from Pakistan (25.2%) [11], Nigeria (44.1%) [29] and India (69.4%) [30]. The differences seen in HCPs knowledge between our study and the previous reports may be because these countries have more developed probiotic industry and healthcare professionals are in more frequent contact with the probiotic product marketing professionals [31].

The present study involved 348 pharmacists, with a gender distribution of 53.6% male and 46.4% female. This gender distribution is comparable to that reported in a study conducted among Jordanian healthcare providers, where a similar male-to-female ratio was observed [1]. Also, it is comparable to findings from a study conducted in Saudi Arabia, where the majority of participants were also male (56.5%) compared to females (43.5%) [15]. However, a study conducted in the UAE showed an opposite trend, with a significantly higher percentage of female pharmacists (63.65%) compared to males (36.4%) [12]. This variation may reflect differences in workforce demographics and gender representation in pharmacy practice across Middle Eastern countries. A significant majority of the participants were aged between 18 and 34 years (76.3%), indicating a youthful cohort. Most participants held a bachelor’s degree (69.3%), which matches the standard educational requirement for entry into the pharmacy profession in many regions. The largest professional roles among the participants were community pharmacists (39%) and clinical pharmacists (16.3%). This distribution reflects the diverse settings in which pharmacists operate, with community pharmacists playing a pivotal role in direct patient care and medication management. The distribution of years of experience varied, with the majority having 0–4 years (59%). This finding is indicative of a relatively inexperienced cohort, which may impact their confidence and decision-making in the practice.

The study found that male pharmacists exhibited a higher percentage of poor knowledge (90.4%) compared to females (87%). This is consistent with findings from other studies, such as those conducted in Europe, where male healthcare professionals often reported lower self-assessed knowledge levels compared to their female counterparts [32]. Males exhibited a higher frequency of negative attitudes (25%) compared to females (10.5%), a finding consistent with a previous study that reported females generally had more positive attitudes while males demonstrated more negative perceptions. This difference may be attributed to the fact that females tend to have a greater interest in dietary supplements and nutrition-related issues [1]. Younger pharmacists aged 18–24 had the highest percentage of poor knowledge (94.4%), which may reflect their limited exposure and experience in the field. A similar trend was observed in the Jordanian study [1], where early-career pharmacists exhibited lower levels of probiotic-related knowledge compared to their more experienced counterparts. Also, this age group also showed the most negative attitude, indicating a potential gap in awareness and acceptance of probiotics. In contrast, pharmacists aged 45–54 and above had 100% positive attitudes. Poor practice was highly prevalent across all age groups, reaching 100% in the 35–44 and ≥ 65 age groups. That may be likely due to limited professional experience and fewer opportunities for continuing education.

This finding is consistent with studies conducted in Jordan, the UAE, and Saudi Arabia. In Jordan, healthcare providers under 30 years old had significantly lower knowledge scores compared to their older counterparts. Similarly, in the UAE, younger pharmacists (22–30 years old) comprised most respondents, yet a substantial proportion exhibited only moderate knowledge of probiotics. In Saudi Arabia, a study on health science students also reported that younger participants had lower confidence in their knowledge and application of probiotics compared to more experienced individuals. These findings highlight the need for targeted educational interventions for young pharmacists to enhance their confidence and competence in probiotic counseling.

Pharmacists with a bachelor’s degree had the highest percentage of poor knowledge (91%) and negative attitude (21.5%), while those with a Ph.D. had the lowest percentage (80% and 0%, respectively). This indicates that higher education is associated with better knowledge and more positive attitudes towards probiotics. Significant differences in attitude (*p* < 0.05) were observed across educational backgrounds. Sales and marketing professionals and clinical pharmacists had the most positive attitudes (92.6% and 94.7% positive, respectively). This could be due to their direct involvement in patient care and health promotion, which necessitates a positive outlook on health interventions like probiotics. Significant differences in attitude were observed (*p* < 0.001), highlighting the influence of professional roles on attitudes towards probiotics. Poor practice was highly prevalent across all professional roles, reaching 100% among researchers and industry pharmacists, indicating a consistently low level of appropriate probiotic-related practice.

Pharmacists with 0–4 years of experience had a high percentage of poor knowledge (93.7%) and negative attitude (22.8%). Significant differences in knowledge (*p* < 0.05) and attitude (*p* < 0.05) were found across years of experience, with those having 10–14 and 15 + years of experience showing a higher percentage of good knowledge (77.8% and 80%, respectively) and positive attitude (89% and 97.5%, respectively). This suggests that experience plays a critical role in enhancing knowledge and shaping positive attitudes towards probiotics.

The analysis of factors associated with good knowledge of probiotics among pharmacists reveals significant insights. Pharmacists aged 45–54 demonstrated a strong likelihood of possessing good knowledge. This suggests that age is a critical factor, potentially due to accumulated experience and exposure to professional development opportunities over time. Experience also emerged as a significant predictor, with pharmacists having 10–14 years of experience. This emphasizes the significance of practical experience in augmenting understanding of probiotics, as pharmacists with greater duration in the profession are more likely to have encountered a broader spectrum of clinical settings and training resources. The analysis of attitudes toward probiotics indicates that gender and age are significant factors. Female pharmacists were 2.8 times more likely to have a positive attitude compared to males. However, after adjustment suggesting that while gender differences exist, they are insignificant when other factors are considered. This finding is in accordance with previous studies indicating that female healthcare professionals often exhibit more favorable attitudes towards health interventions [32].

Age also played a significant role, with younger pharmacists aged 25–34 showing a strong association with positive attitudes. This suggests that younger pharmacists may be more open to adopting new health practices, possibly due to recent educational experiences that emphasize the benefits of probiotics although that a previous study did not find any relation between age of practitioners and knowledge, attitude or practice towards probiotics [11]. Professional roles further influenced attitudes, with pharmacists in sales and marketing and clinical pharmacists showing significant positive attitudes. These roles likely involve more direct patient interaction and a focus on health promotion, which may enhance their perception of probiotics’ benefits. The analysis of poor practice regarding probiotics revealed that pharmacists aged 45–54 were significantly associated with poor practice. However, the AOR was unstable (reported as 0) (95% CI not estimable, *p* ≥ 0.05), likely due to sparse data rather than indicating no association. Pharmacists with 15 + years of experience also showed a significant association with poor practice, but the AOR was an outlier, likely due to the small sample size. These findings suggest that while age and experience are important, other factors may also influence practice behaviors. The high prevalence of poor practice across various demographics indicates a gap in appropriate probiotic-related practice, although further research is needed to explore the underlying factors driving these practices.

As highlighted in the results, the main barriers to probiotic recommendation included a lack of established clinical applications and insufficient knowledge. These findings align with previous studies, emphasizing the barriers related to counselling about DHSs in general [33]. Initially, pharmacists have insufficient understanding of dietary herbal supplements, particularly concerning the interactions between medications and these supplements. It’s important to highlight that this deficit in knowledge is primarily due to the scant education and training on DHSs that pharmacists receive during and after their formal studies, which has undermined their self-assurance in offering advice and added to the uncertainty about their professional duties. The absence of dependable resources and adequate time were frequently cited as significant obstacles to provide counseling. To address this, it is imperative that high-quality resources detailing the safety and effectiveness of dietary herbal supplements be made readily available to pharmacists [33]. This aligns with the current study’s findings, where the majority of pharmacists cited this as their primary concern. While the lack of established applications was the most frequently mentioned barrier, a smaller group of pharmacists identified insufficient knowledge on the clinical use of probiotics as a concern. This highlights a gap in education and training, which is crucial for the effective integration of probiotics into healthcare practices. Therefore, we encourage professional organizations to support pharmacists in overcoming barriers related to probiotics through various initiatives, including continuing education and training programs, the development of evidence-based guidelines, and the creation of probiotic reference materials such as clinical handbooks, mobile applications, and online databases to aid decision-making. Additionally, collaborating with universities to integrate probiotics education into undergraduate and postgraduate pharmacy programs, launching public awareness campaigns and patient education tools, conducting community outreach programs on the appropriate use of probiotics, and facilitating collaborative learning opportunities between pharmacists, physicians, and dietitians can help bridge the knowledge gap between healthcare professionals and patients while promoting a multidisciplinary approach to probiotic recommendations.

In conclusion, despite the increasing popularity of probiotics, the knowledge, attitudes, and practices of pharmacists regarding probiotics remain crucial yet understudied factors. Given the pivotal role pharmacists play in health education and promotion, understanding their familiarity with and perspectives on probiotics is essential. This research showed the importance of ongoing education and training for pharmacists to optimize patient care in probiotic interventions.

### Limitations

Small sample size in certain subgroups such as the number of elderly pharmacists (aged > 65 years) participating in the survey was limited which may affect the generalizability of the findings to this age group and the small proportion of pharmacists working in the pharmaceutical industry, which may not fully capture their perspectives on the topic. In addition, conducting the survey online may have introduced selection bias, as participation was limited to those with internet access and willingness to engage in digital surveys. Also, the survey did not include specific questions regarding the pharmacists’ primary sources of medicine-related information, which could have provided valuable insights into their decision-making processes.

These limitations highlight the need for further studies with a broader and more diverse sample, incorporating different data collection methods.

## Supplementary Information

Supplementary Material 1.

## Data Availability

The data supporting this study’s findings are available from the corresponding author on a reasonable request.
