# Supplementary material for: Assessing the knowledge, attitude, and practice of Egyptian pharmacists on probiotics: a cross-sectional study
Source: BMC Complement Med Ther. 2026 May 25;26:194. doi: 10.1186/s12906-026-05406-2 (PMC13200401; doi:10.1186/s12906-026-05406-2)
Supplement: Supplementary file 1 — Supplementary Material 1. [file 12906_2026_5406_MOESM1_ESM.pdf]

Assessment of Pharmacists' Knowledge, Attitudes, and Practices Regarding Probiotics.

1.

We cordially invite you to take part in an online questionnaire designed to evaluate pharmacists' knowledge, attitudes, and practices concerning probiotics.
Please be assured that the information you provide will be treated with the utmost confidentiality. The survey should require no more than five minutes of your time.
Thank you for your valuable contribution.

* 1. What is your Gender?

- ◯ Male
- ◯ Female

* 2. What is Your Age?

- ◯ 18-24
- ◯ 25-34
- ◯ 35-44
- ◯ 45-54
- ◯ 55-64
- ◯ 65+

* 3. Educational Background

- ◯ Bachelor’s degree
- ◯ Post graduate diploma
- ◯ Master’s degree
- ◯ PhD
- ◯ Other (please specify)

* 4. What is your current professional role in the pharmacy field?

- ◯ Hospital Pharmacist
- ◯ Academic staff
- ◯ Sales and Marketing
- ◯ Research
- ◯ Industry
- ◯ Community Pharmacist
- ◯ Senior pharmacy student (Internship year)
- ◯ Clinical pharmacist
- ◯ Other (please specify)

* 5. How many years of experience do you have in your current professional role?

- ◯ 0-4
- ◯ 5-9
- ◯ 10-14
- ◯ ≥15

Assessment of Pharmacists' Knowledge, Attitudes, and Practices Regarding Probiotics.

2. Knowledge

* 6. Could you please indicate which one of the following definitions you believe is the most accurate?

- ◯ Probiotics are live organisms (mostly bacteria) that are helpful to your health when you eat them.
- ◯ Probiotics are diet products for the good bacteria in your body.
- ◯ Probiotics are natural antibiotics.
- ◯ Probiotics are chemicals to help kill bacteria on fruit and vegetables.
- ◯ Probiotics are substances that make food taste sweeter.

* 7. Could you please check the boxes next to the strains of probiotics that you recognize or have knowledge of? Please check all that apply

- [ ] Lactobacillus
- [ ] Bifidobacterium
- [ ] Saccharomyces
- [ ] Streptococcus
- [ ] Enterococcus
- [ ] Escherichia coli
- [ ] Bacillus

* 8. Could you please provide your perspective on whether probiotics are beneficial for the following health conditions? Please check all that apply

- [ ] Acute Diarrhea/ Antibiotic-associated diarrhea
- [ ] Allergies
- [ ] Atopic dermatitis
- [ ] Clostridium difficile Infections
- [ ] Crohn’s disease
- [ ] General digestion/gut health
- [ ] Heart health
- [ ] Hypercholesterolemia
- [ ] Immune health
- [ ] Irritable bowel syndrome
- [ ] Mental health
- [ ] Overweight/obesity
- [ ] Periodontal diseases and halitosis
- [ ] Prevention of Respiratory Infections
- [ ] Prevention of Urinary Tract infections
- [ ] Ulcerative colitis
- [ ] Urinary tract infection
- [ ] Urogenital conditions

* 9. Could you please select all the dietary sources of probiotics that you are aware of from the following list? Please check all that apply

- ☐ Yogurt
- ☐ Cheese
- ☐ sourdough Bread
- ☐ Chocolate
- ☐ Olives
- ☐ Pickles
- ☐ Soy based products
- ☐ Cereals
- ☐ Cabbage
- ☐ Breast milk

* 10. Do you know whether probiotics exerts a beneficial effect without colonizing the site?

- ◯ Yes
- ◯ No
- ◯ I do not know

* 11. Are you aware of commercially available forms of probiotics?

- ◯ Yes
- ◯ No
- ◯ I do not know

* 12. Are probiotics safe for pregnant women?

- ◯ Yes
- ◯ No
- ◯ I do not know

* 13. There are minimal risks associated with the clinical use of probiotics for most patients.

- ◯ Yes
- ◯ No
- ◯ I do not know

* 14. Probiotics have the potential to upset the usual equilibrium of intestinal microbes.

- ◯ Yes
- ◯ No
- ◯ I do not know

* 15. The production of anti-inflammatory cytokines and antibodies can be increased by probiotics.

- ◯ Yes
- ◯ No
- ◯ I do not know

Assessment of Pharmacists' Knowledge, Attitudes, and Practices Regarding Probiotics.

3. Attitude

* 16. Do you consider probiotics are good for human health?

- ◯ Not at all
- ◯ Some what
- ◯ Very much

* 17. Do you consider that probiotics can be dangerous for health?

- ◯ Not at all
- ◯ Some what
- ◯ Very much

* 18. If we provide you proper reference about probiotics use, would you be willing to recommend probiotics to your patients?

- ◯ Not at all
- ◯ Some what
- ◯ Very much

Assessment of Pharmacists' Knowledge, Attitudes, and Practices Regarding Probiotics.

4. Practice

* 19. In your professional practice, would you recommend probiotics for any of the following health conditions? Please check all that apply

- [ ] Allergies
- [ ] Atopic dermatitis
- [ ] Clostridium difficile Infections
- [ ] Crohn’s disease
- [ ] General digestion/gut health
- [ ] Heart health
- [ ] Hypercholesterolemia
- [ ] Immune health
- [ ] Irritable bowel syndrome
- [ ] Mental health
- [ ] Overweight/obesity
- [ ] Periodontal diseases and halitosis
- [ ] Prevention of Respiratory Infections
- [ ] Prevention of Urinary Tract infections
- [ ] Ulcerative colitis
- [ ] Urinary tract infection
- [ ] Urogenital conditions

* 20. I believe that probiotics could serve as an alternative approach to prevent digestive system symptoms caused by pathogenic infections or diseases.

- ○ Yes
- ○ No

* 21. I see potential in probiotics for modifying the side effects of antibiotics.

- ○ Yes
- ○ No

* 22. As a healthcare provider, I would promote the use of probiotics for both the prevention and treatment of digestive system infections.

- ○ Yes
- ○ No

* 23. I am interested in learning more about probiotics.

- ○ Yes
- ○ No

* 24. I would appreciate opportunities for education or workshops that focus on the various uses of probiotics.

- ◯ Yes
- ◯ No

* 25. I am open to the concept of using live organisms as part of the strategy for managing medical conditions.

- ◯ Yes
- ◯ No

* 26. I recommend probiotics without any concerns.

- ◯ Yes
- ◯ No

Assessment of Pharmacists' Knowledge, Attitudes, and Practices Regarding Probiotics.

5. Barriers to recommend probiotics

* 27. What might be the reasons for not recommending probiotics in your professional practice? (Please select all that apply)

- [ ] Probiotics have no established clinical applications within my area of expertise.
- [ ] I am not convinced of the clinical benefits of probiotics.
- [ ] The cost of probiotics is a deterrent.
- [ ] I have had negative experiences with the use of probiotics in the past.
- [ ] I lack sufficient knowledge about the clinical use of probiotics.
